# The role of psychological resilience and aggression in injury prevention among martial arts athletes

**DOI:** 10.3389/fpsyg.2024.1433835

**Published:** 2024-06-26

**Authors:** Ionuț Patenteu, Roman Gawrych, Mircea Bratu, Luciela Vasile, Ryszard Makarowski, Andrei Bitang, Sarah Adriana Nica

**Affiliations:** ^1^Faculty of Medicine, University of Medicine and Pharmacy “Carol Davila”, Bucharest, Romania; ^2^University of Social and Economics in Gdańsk, Gdańsk, Poland; ^3^Special Motricity and Medical Recovery Department, Faculty of Physiotherapy, National University of Physical Education and Sports, Bucharest, Romania; ^4^Doctoral School Department, Faculty of Physical Education and Sport, National University of Physical Education and Sports, Bucharest, Romania; ^5^Faculty of Administration and Social Sciences, Academy of Applied Medical and Social Sciences in Elblag, Elblag, Poland; ^6^University of Social and Economics in Gdańsk, Gdańsk, Poland; ^7^Faculty of Physical Education and Sport, "Aurel Vlaicu" University of Arad, Arad, Romania

**Keywords:** resilience, sports injury, martial arts, aggression, assertiveness

## Abstract

**Introduction:**

For martial artists, the ability to manage reactions in the face of adversity and bounce back after a stressful event can have major impact on performance. The scope of the research is to investigate martial artists’ level of resilience and aggression (Go-ahead, Foul play, and Assertiveness factors), what is specific to athletes and who have suffered from moderate and/or severe injuries (in terms of resilience and three factors of aggression examined), and test the possibility that a psychological variable under investigation can predict athletes’ injury severity.

**Materials and methods:**

A total sample of 154 athletes from striking combat sports—SC (karate, taekwondo, kickboxing, and boxing), grappling combat sports—GC (judo and BJJ), and mixed martial artists (MMA) participated in the research. For assessing resilience, the Romanian adaptation of the Brief Resilience Scale (BRS) was used, and for aggression, the Romanian adaptation of Makarowski’s Sports Aggression Questionnaire was used. An injury report form was also created and applied to athletes.

**Results:**

The post-hoc tests (after running a single-factor multivariate analysis of variance) revealed significant differences for resilience and Foul (violent) play between the sports disciplines analyzed. A significant positive correlation was found between athletes’ injury severity and assertiveness in SC and between injury severity and resilience in GC. Through the *t*-test for independent samples, it was highlighted that the average value for Foul (violent) play is significantly higher in athletes who have suffered mild, moderate, and/or severe injuries compared with martial arts athletes who have suffered from only mild/minor injuries. A binomial logistic regression was also performed to verify to what extent Foul play predicts athletes’ injury severity.

**Conclusion:**

A low level of Foul (violent) play is linked with a decreased likelihood of moderate and/or severe injuries in martial arts athletes. The study findings suggest that resilience, foul play, and assertiveness have an important role in injury prevention among martial artists.

## 1 Introduction

As heuristic sport disciplines and direct contact with the opponent, martial arts require fast decisions under stressful situations, mental toughness, and creativity under un-certainty (Predoiu et al., 2018). Researchers underlined the importance of practicing martial arts to promote ethics, moral values, and social development of practitioners (Fukuda et al., 2011; Kostorz and Sas-Nowosielski, 2021a). However, there may be times when the win-at-all-costs philosophy (way of thinking that can manifest regardless of age and level of training) can facilitate the manifestation of hostile aggression and violence (Urzeală and Teodorescu, 2018), making sports injuries more likely.

Martial arts can be divided into Striking Combat sports—SC (e.g., karate, boxing, Muay Thai, kenpō, kick-boxing, and taekwondo), Grappling Combat sports—GC (e.g., judo, freestyle wrestling, and BJJ), as in previous study (Predoiu et al., 2022a), while Mixed Martial Arts (MMA) involve techniques from both striking and grappling fighting styles (Miarka et al., 2019). In addition, Kalina (2000) argued that “every combat sport is martial arts but not vice versa” (p. 18).

A question that has aroused the interest of specialists relates to the association between martial arts athletes’ injury severity and their level of sports performances. For example, during low-level competitions, a higher frequency of injuries was reported (Frey et al., 2004). However, in a recent study, authors found no significant link between the two variables (martial arts athletes were investigated separately, from SC, GC, and MMA) (Patenteu et al., 2023). Interestingly, in 2012, Kazemi argued that in elite taekwondo athletes, injuries before competition were related to a 30% increase in medal prevalence (although not statistically significant) (Kazemi, 2012).

Considering the definition of sports injuries, we will present the following: “damage or a wound caused to a person’s body” (Dictionary of Sport and Exercise Science, 2006); “any sports-related musculoskeletal complaint that resulted in an athlete to stop, limit, or modify participation for one or more days” (Li et al., 2015). After exploring the existing data in the literature (Caine and Maffulli, 2005; Piedade et al., 2021), we may conclude that in the case of minimum 2–3 weeks missed/time loss from training or competition, we can discuss about (at least) a moderate injury. Injuries are very important stressors encountered by athletes, being classified in the category (of stressors) linked to competitive performance (Sarkar and Fletcher, 2014). Therefore, on the road to great sports results, athletes’ resilience/ability to positively cope with difficult events (and overcome them) is essential.

Coming from mammalian adaptation literature, resilience (as a basic definition) refers to an organism’s ability to adapt to one’s life-preserving capability when confronted to stressful conditions (Kent et al., 2013). In the case of humans, psychological resilience represents the mental ability that helps people withstand emotional, cognitive, or physical difficulties (Everly et al., 2015), being a psychological phenomenon that operates at multiple levels (Masten et al., 2021). In their systematic review of resilience across sports and work, after conducting a frequency word analysis, researchers found that most definitions included concepts such as bouncing back (cf. rebound), positive adaptation, and maintenance of well-being (in face of adversity) (Bryan et al., 2019). Resilience is not only the ability to bounce back from failure or challenges but also the ability to be stronger (mentally or physically) and obtain superior results in the task performed (Fletcher and Sarkar, 2013). Therefore, resilience facilitates learning, development, recovering and growth (Shi et al., 2019), authors discussing about, growth following adversity in competitive sport” (Howells et al., 2017).

According to the sporting resilience meta-model (Gupta and McCarthy, 2022), the biopsychosocial protective filter makes its mark on adversity, determining a lower or a higher disruption in athletes. More resilient athletes will return faster to their sports performance (after a disruption), while less resilient athletes (having fewer biopsychosocial resources to bounce back after failure) are likely to experience negative sports results followed by further negative performances in competitions (Bejan-Mureșan and Cinpeanu, 2019). Moreover, considering the grounded resilience theory (Fletcher and Sarkar, 2012), protective factors such as perceived social support, confidence, motivation, focus, and positive personality (resources that are influenced by challenge appraisals and metacognitions) contribute to resilience in successful athletes. In sports field, psychological variables associated with maintaining or building resilience were identified, the most important being (Bryan et al., 2019) as follows: self-efficacy, coping skills, support, perspective, motivation, optimism, self-regulation, hardiness, and positive mindset.

Social support and coping skills must interact to increase resilience and reduce distress associated with sports injuries (Smith et al., 1990). Investigating athletes with spinal cord injury, researchers highlighted multiple types of social support, behavioral and cognitive coping strategies, and motivation to adapt as important resources to increase athletes’ resilience (Machida et al., 2013). Galli and Gonzalez (2015) mentioned that only recently specialists and practitioners have begun to investigate the construct of resilience within the sport environment. Psychological resilience was examined between current and former high-level athletes (Galli and Vealey, 2008) and various competitive athletes, for example, swimmers (Morgan et al., 2013), handball and soccer players, or futsal teams (Mummery, 2008). In a recent article, resilience level of athletes (including martial artists from striking combat sports—boxing, karate, fencing, kickboxing, taekwondo, and grappling combat sports—judo) was investigated in relation to the Big Five personality factors (Rawat et al., 2023).

With respect to martial artists, resilience is positively influenced by athletes’ schooling level and practice time and affected by psychoticism, in this last situation athletes being less likely to experience high degrees of resilience (da Gama et al., 2018). Psychological resilience has the potential to mitigate the negative effect of distress and exhaustion in martial arts athletes (judokas were examined) (Jo, 2016), while in the case of taekwondo, adolescent practitioners contribute to performance, more exactly, “psychological skills work positively between resilience and performance” (Yang et al., 2019). When talking about preadolescents, martial arts-based interventions significantly increased resilience level (Moore et al., 2021).

It was found that psychological resilience is an essential factor linked with recovery time (and not only) following sports-related concussions: low resilience values predict protracted recovery (Ernst et al., 2022) and were related to higher scores for depressive symptoms and anxiety during recovery (Bunt et al., 2021). Resilience was also linked with adjustment and adaptation for persons faced with acquired brain injury (Neils-Strunjas et al., 2017), while positive reframing, optimism, athletes’ informational social support, and injury centrality represent predictors for sport injury-related growth (Pollak et al., 2022). There is a gap in the literature considering the resilience level of martial arts athletes (also, investigated separately from SC, GC, and MMA) and its link with athletes’ injury severity in competitions.

The second psychological variable examined in the present study in relation to martial arts athletes’ injury severity is explicit aggression (measured primarily through questionnaires), different from implicit aggression, which is observed as a “result of automated processes that can be assessed with indirect measurement tools (e.g., Implicit Association Test)” (Predoiu et al., 2022b). Researchers present aggression as a person’s behavior aimed at causing pain (the stimulus could be physical, a gesture, or verbal) (Klimczak et al., 2014). Therefore, by definition, aggression supposes the intent to produce harm/damage to another person. In these conditions, it is probable that the incidence of injury in a specific sports discipline to be linked to the amount of aggression involved in that sports. For example, rugby, hockey, soccer, MMA, and other contact sports “are likely, by their very nature, to produce injuries, whereas sports that are less aggressive such as golf and tennis are less likely to do so” (Pedersen, 2007). These results were confirmed by Pedersen (2007) that the probability of a severe injury is the highest in football, followed by boxing, hockey, rugby, gymnastics, and wrestling (16 sports branches were investigated).

However, in sports, aggression has a positive connotation (Cashmore, 2008), most of the time being instrumental (see, for example, Silva for instrumental and hostile aggression—Silva, 1983), athletes manifesting it to achieve the objective set (e.g., to win/dominate in a competition or to score in a certain situation) (Predoiu et al., 2022a). The level of training or training duration cannot significantly influence the degree of aggression in martial artists (Kostorz and Sas-Nowosielski, 2021b). Anger, verbal reactions, dominance behavior during competition, and physical violence are all forms in which aggression can manifest. To win inevitably, martial arts athletes must act aggressively, but this type of aggression is accepted socially (in the context of respecting the rules of the game) (Vít et al., 2019).

Following a systematic review which addressed psychological factors that influence the severity of sports-related concussions (Trinh et al., 2020), a gap considering the aggression level of martial artists in relation to injury severity persists. For example, a higher level of aggression and negative emotionality were related to an increased incidence of sports-related concussions (football players were examined in this case) (Klotz et al., 2017). However, other authors underlined that footballers’ aggression has less influence on their head impacting exposure as compared with other factors (Marks et al., 2022). In addition, aggressive tennis players, exposing more in competitions, are more likely to get injured (Aggression scale of the Factor Personality Inventory—FPI was used) (Keller et al., 2013). In the case of anger (a factor of aggression, see Buss and Perry, 1992), it was linked with higher incidences of athletic injuries (Galambos et al., 2005), but no combat sports athletes were included in the sample. This is particularly important that in martial arts, a higher level of anger facilitates sports performance (Terry and Slade, 1995; Wargo et al., 2007), an aspect that cannot be asserted when talking about other sports disciplines (Maxwell et al., 2009).

In the current study, we investigated the following three factors of aggression: Go-Ahead, Assertiveness, and Foul Play, with respect to Ryszard Makarowski’s model (Makarowski et al., 2021). Foul (violent) play is the closest to the definition of aggression presented in the current study (see Klimczak et al., 2014), referring to the manifestation of violent game strategies to harm and block the opponent, even in an unethical manner (including “dirty” play). Go-Ahead factor refers to the perseverance in achieving objectives despite numerous obstacles. Athletes reach their goals, sometimes regardless of the costs (they tend to observe obstacles as challenges). Regarding assertiveness in sports, it is the ability to behave decisively within appropriate boundaries, to voice criticism (critical feedback), and to express emotions and thoughts directly and firm. An assertive athlete respects the rules in competition which can lead to success (Bredemeier, 1994).

Therefore, the purpose of the current research was to investigate the level of resilience and aggression (Go-Ahead, Foul play, and Assertiveness) in competitive martial arts athletes, according to the practiced sports discipline. In the present study, we refer to grappling combat sports, striking combat sports, and striking and grappling combat sports (Mixed Martial Arts, which combines techniques from both fighting styles), as in previous studies (Predoiu et al., 2022b; Patenteu et al., 2023). At the same time, we wanted to capture the differences between martial arts athletes who have suffered at least medium severity injuries (minimum 2–3 weeks missed/time loss from training or competition) and athletes who have not suffered such sports injuries up to the time of testing (in terms of resilience and aggression). Not least, we verified whether the resilience level and the aggressive behavior can predict injury severity in martial artists.

The present research is part of a broader study, being a continuation of research aimed at identifying psychological variables that predict injury severity in martial arts (see Patenteu et al., 2023, where trait anxiety and risk-taking behavior were investigated in relation to martial arts athletes’ injury severity).

### Objectives

- Establishing the level of resilience and aggression in martial artists;
- Knowing the links between athletes’ level of aggression, resilience, and martial arts athletes’ injury severity;
- Identifying the differences between athletes, in terms of resilience and aggression, taking into consideration martial artists’ injury severity;
- Knowing predictors of injury severity in the case of martial arts athletes.

In the current study, it was hypothesized that:

- There are significant differences between martial arts athletes according to the specifics of the sports disciplines (striking combat sports, grappling combat sports, respectively, striking and grappling—MMA), in terms of resilience and aggression;
- There are significant correlations between athletes’ injury severity and martial arts’ level of resilience and aggression.
- Investigation of martial arts athletes who have suffered mild, moderate, and/or severe injuries and athletes who have suffered only minor/mild injuries reveals significant differences between the two groups in terms of resilience level and aggressive behavior.
- The results for aggression represent a predictor of injury severity among martial arts athletes.

## 2 Materials and methods

### 2.1 Participants and procedure

One hundred and fifty-four Romanian competitive martial arts athletes, affiliated at different sports clubs in Romania, male (*n* = 132) and female (*n* = 22), aged between 20 and 32 years, were participated in the study. The participants are the same martial arts athletes involved in the previous study (Patenteu et al., 2023). The questionnaires for assessing resilience and aggression were applied via Google forms (Google LLC, Mountain View, CA, United States) between March 2022 and September 2022. To clearly understand, the characteristics of the participants (and reduce the repetitive nature of data related to sample characteristics) and the descriptive statistics of surveyed martial arts and combat sports athletes can be accessed through the following hyperlink— Descriptive statistics of surveyed martial arts athletes (Patenteu et al., 2023).

The following sample characteristics are the same as in the previous study: competitive experience (M = 8.39, SD = 3.10—in the entire sample); inclusion criteria—(a) athletes having minimum 20 years old, (b) minimum 4 years of experience in competitions, (c) minimum 12 official fights/year, and (d) without severe injuries prior to the examined period (January 2018–December 2021). We mention that 19 athletes were removed from the research following the last inclusion criteria (from the 188 athletes who completed the questionnaires in the initial phase of the research), the reasoning behind this decision being related to the possible fear of reinjury (Cassidy, 2006), or anxiety associated with return to play (Bennett and Lindsay, 2016). These challenges (for martial artists), when returning from serious injuries, can affect future behaviors during competitions. Therefore, “a relatively similar level in terms of the severity of injuries suffered by athletes at time *t*_0_” (Patenteu et al., 2023) was assured.

On the other hand, 15 martial arts athletes were removed from the study due to the (c) inclusion criteria. We mentioned that during the pandemic, “martial arts competitions were organized in Romania (and televised) but without spectators. Only athletes and coaches had access in the competition hall, and they were previously tested against COVID-19” (Predoiu et al., 2022a) (each martial arts athlete who participated in the study had a minimum of 12 official fights/year during the first year of the COVID-19 pandemic). The mean, at group level, was 16.8 matched per year (SD = 1.79).

In the study by Li et al. (2015), in the situation of a recurrent injury, it was counted once (this is the case of 16 athletes, who had the same moderate injury twice). In addition, in the case of 47 martial artists who suffered from a severe injury (30.5% of participants), the minimum 12 fights/year (inclusion criteria c) were counted from the first official match after recovery (because these athletes, following the severe injury, were out from competitions for months). Injuries were counted in the last 4 years (during January 2018–December 2021), being a retrospective study. The snowball sampling technique was used, as in the previous research (Patenteu et al., 2023).

### 2.2 Measures

Aggression was measured with the Romanian adaptation of the Makarowski’s Sports Aggression Questionnaire (Makarowski et al., 2021) (the norms are the same for male and female martial arts athletes). It consists of 15 items arranged into three subscales (five items for each subscale): Foul play (e.g., I think that “anything goes” rule is appropriate to achieve the victory), Assertiveness (e.g., I’m not afraid to speak up to my supervisor or coach, if I know that he/she is wrong), and Go-Ahead (e.g., There is no argument that would turn me away from reaching my goal). The martial artists indicated their responses using a five-point Likert-type scale, where “a” = Definitely not (1 point), “e” = Definitely yes (5 points), “c” = Hard to say (3 points), “b” = Probably not (2 points), and “d” = Rather yes (4 points). In the current research, reliability for the three factors/subscales, measured with McDonald’s omega coefficient (*ω*), was 0.71 (Go-Ahead), 0.74 (Foul Play), 0.75 (Assertiveness), respectively.

To assess resilience, the Romanian adaptation of the Brief Resilience Scale (BRS) was used (Alexe et al., 2022). BRS consists of six items to which martial arts athletes answered by choosing a response option from 5 to 1, where 5 = Total Agreement and 1 = Total Disagreement. The questionnaire has three reverse scoring items. The higher the values obtained, the higher the degree of athletes’ resilience. Item example:” I tend to bounce back quickly after hard times” (Smith et al., 2008). In the Romanian adaptation, BRS” revealed that adequate fit-indexes” and” suitable values were also obtained for reliability and convergent validity” (Alexe et al., 2022). In the current research, McDonald’s *ω* reliability coefficient was 0.78. BRS was used in previous research on martial artists (Kuçuk Kiliç, 2020).

The injury report form (consisting of 10 close- and open-ended questions) realized starting from investigation by Willick et al. (2013), and the results obtained are the same as in the previous research (Patenteu et al., 2023) because the same martial artists were examined as explained at *Participants and Procedure* subsection. Therefore, data regarding age, gender, sports discipline practiced, competitive experience, and the highest sports performance obtained were gathered, as well as data regarding the number of official fights/year (January 2018–December 2021) and athletes’ injury severity (examples of types of injuries were given to be selected, and also, blank spaces where athletes could fill in the type of injury suffered). Not least, martial arts athletes were asked about a severe injury suffered before January 2018 (response options Yes/No—question related to the inclusion criterion d). Athletes in this research suffered moderate or severe injuries only in competitions/official matches (however, mild/minor injuries were suffered also inevitably during training and competition).

The Desirability Scale of the Zuckerman-Kuhlman Personality Questionnaire (ZKPQ) was also used. Following the application of this scale [ZKPQ is calibrated on the Romanian population (Miclea et al., 2009)], no athlete was removed from the study (no martial arts athlete exceeds the threshold set by the Desirability Scale), meaning that the responses were not distorted and the likelihood of inappropriate answers was very low.

### 2.3 Research design

The investigation is based on *ex post facto* design—the analysis started (the online surveys were applied) after the fact has occurred, martial art athletes already suffered certain injuries (between January 2018–December 2021) and obtained various sports results in competitions, without interference from the researchers (is a retrospective research).

### 2.4 Statistical analysis

First, using stem-and-leaf and boxplots, data were screened for outliers (Tabachnick et al., 2013). Then, descriptive statistics were used through means and standard deviation. After that, MANOVA was used to see whether the results for resilience and the three factors of aggression differ significantly according to the practiced sports discipline. Because Box M is insignificant, we therefore refer to Wilk’s Lambda test value, while according to Levene’s test results, Scheffe post-hoc test (when equality of variance was assured—*p* > 0.05) and Tamhane post-hoc test (when *p* < 0.05), respectively, were interpreted (Popa, 2010). Then, Pearson correlation was used to verify the associations between variables, with the effect size index (coefficient of determination—*r*^2^) interpretation: 0.25 (a large effect), 0.09 (a moderate effect), and 0.01 (small effect) (Cronk and Cronk, 2020). In case of the Independent Samples *t*-test, the normality of the distributions was checked using the skewness coefficient, this value being less than 1 (Morgan et al., 2004). Data analysis also involved the use of binomial logistic regression, Nagelkerke *R*^2^ effect size having the following interpretation: 0.2 small, 0.15 medium effect size, and 0.35 large (Cohen, 1992). IBM SPSS Statistics 27.0 (Armonk, NY, IBM Corp) and Jamovi (for calculating McDonald’s omega reliability coefficients) were used.

## 3 Results

Table 1 shows the descriptive statistics (in the case of resilience and the three factor of aggression) by sports discipline: striking combat sports (*n* = 64), grappling (*n* = 39), and MMA (*n* = 51).

**Table 1:** Descriptive statistics by sports discipline (*n* = 154).

Variables	Sport disciplines	Results	
Resilience	SC	Mean	3.7159
		SD	0.53181
	GC	Mean	3.4333
		SD	0.53214
	SC and GC (MMA)	Mean	3.7710
		SD	0.58425
Go-ahead	SC	Mean	17.83
		SD	3.757
	GC	Mean	16.90
		SD	3.538
	SC and GC (MMA)	Mean	17.65
		SD	3.230
Foul Play	SC	Mean	8.03
		SD	3.285
	GC	Mean	6.72
		SD	2.051
	SC and GC (MMA)	Mean	8.61
		SD	3.595
Assertiveness	SC	Mean	17.53
		SD	4.309
	GC	Mean	18.10
		SD	3.648
	SC and GC (MMA)	Mean	18.96
		SD	3.939

SC, Striking combat sports; GC, grappling combat sports; MMA, mixed martial arts.

The analyses in Table 1 show (in the case of the investigated martial artists and at group level) a moderate level of resilience, a slightly below average level for Go-ahead factor, a low level of Foul (violent) play, and an average level of Assertiveness (according to the norms).

Through one-way MANOVA, we verified the significant differences between martial artists from SC, GC, and MMA, in terms of resilience and aggressive behavior. The linearity condition was ensured (for the multivariate analysis of variance procedure); the correlations were observed between the investigated variables being weak and very weak. Box M test value is 0.064 (insignificant); therefore, Wilk’s Lambda test value was reported: *F*(8, 296) = 2.701, *p* = 0.007, Wilk’s Lambda = 0.869. With respect to the Test of Between-Subjects Effects, the sports discipline significantly influences the results for resilience (*F* = 4.683, *p* = 0.011, Partial Eta Squared = 0.058) and Foul play (*F* = 4.122, *p* = 0.018, Partial Eta Squared = 0.052) (see Table 2).

**Table 2:** Results for the post-hoc tests single-factor multivariate analysis of variance.

DVs		(I) Discipline	(J) Discipline	MD (I-J)	*p*-value
Resilience	Scheffe	SC	GC	0.2826	0.043*
			SC and GC (MMA)	−0.0550	0.868
		GC	SC	−0.2826	0.043*
			SC and GC (MMA)	−0.3376	0.017*
		SC and GC (MMA)	SC	0.0550	0.868
			GC	0.3376	0.017*
Go-ahead	Scheffe	SC	GC	0.93	0.434
			SC and GC (MMA)	0.18	0.963
		GC	SC	−0.93	0.434
			SC and GC (MMA)	−0.75	0.609
		SC and GC (MMA)	SC	−0.18	0.963
			GC	0.75	0.609
Foul Play	Tamhane	SC	GC	1.31	0.042*
			SC and GC (MMA)	−0.58	0.758
		GC	SC	−1.31	0.042*
			SC and GC (MMA)	−1.89	0.007*
		SC and GC (MMA)	SC	0.58	0.758
			GC	1.89	0.007*
Assertiveness	Scheffe	SC	GC	−0.57	0.784
			SC and GC (MMA)	−1.43	0.171
		GC	SC	0.57	0.784
			SC and GC (MMA)	−0.86	0.607
		SC and GC (MMA)	SC	1.43	0.171
			GC	0.86	0.607

SC, striking combat sports; GC, grappling combat sports; MMA, mixed martial arts; MD, mean difference, *p < 0.05.

In the case of Foul play, considering homogeneity of variances, *p* < 0.05 (Levene’s test), the Tamhane post-hoc test was interpreted, while Scheffe test results were presented for the other dependent variables (DVs).

The next step was to check the existing associations between injury severity and athletes’ level of resilience and aggression.

With respect to martial artists’ injury severity: 4 = athletes suffered 1 or 2 moderate injuries +1 severe injury, 3 = martial artists suffered only 1 severe injury, 2 = athletes had (during the investigated period) 1 or 2 moderate injuries, and 1 = only mild/minor injuries. Table 3 contains only the significant correlations highlighted.

**Table 3:** Pearson correlation between resilience, aggression, and athletes’ injury severity.

Injury severity			Resilience	Go-ahead	Foul play	Assertiveness
Striking combat sports						
Assertiveness	*r*	0.411^*^				
	*p*	0.019				
Go-ahead	*r*				0.519^**^	
	*p*				0.002	
Grappling combat sports						
Resilience	*r*	0.451^*^				
	*p*	0.031				
Go-ahead	*r*	0.465^*^ 0.025			0.552^**^	
	*p*				0.006	
Striking and Grappling (MMA)						
Go-ahead	*r*		0.660^***^		0.540^**^	
	*p*		<0.001		0.001	

*p < 0.05, **p < 0.01, and ***p < 0.001.

Table 3 underlines a significant link between injury severity and assertiveness (in striking combat sports), respectively, and between injury severity and resilience (in grappling combat sports). A higher assertiveness score is associated with a higher injury severity in SC (the effect size *r*^2^ = 0.17), and a higher resilience score (athletes recover more quickly after a difficulty) is associated with a higher injury severity in GC (*r*^2^ = 0.20). Regarding the confidence interval (95%), lower limit = 0.184 and upper limit = 0.596 (SC), while lower limit = 0.158 and upper limit = 0.671 (GC).

In addition, the results (Table 3) highlight a positive correlation between Foul (violent) play and Go-ahead, in the case of each sport category: SC, GC, and MMA. As the athlete scores are higher on Go-ahead factor, this is linked with a higher probability of violent behaviors in competition (athlete may break the rules and, therefore, could lose points or could even be disqualified). In addition, a positive correlation was found between resilience and Go-ahead scores in both grappling combat sports and MMA.

To verify the third hypothesis, Independent Samples *t*-test was used (DVs were normally distributed, the skewness coefficient, in absolute value, being less than 1).

Data in Table 4 emphasize that the average value for Foul (violent) play is significantly higher [*t* = 4.20, *p* < 0.01] in athletes who have suffered mild, moderate, and/or severe injuries (M = 8.28, SD = 2.15), compared with martial arts athletes who have suffered only mild/minor injuries (M = 6.93, SD = 1.73). The effect size index (Hedge’s *g*) is 0.66, meaning a moderate to strong difference between the results.

**Table 4:** Athletes who have suffered mild, moderate, and/or severe injuries (*n* = 87) and athletes who have suffered only mild/minor injuries (*n* = 67).

DV’s	Levene’s test F *p*		*t*	df	*p*-value	Confidence interval	
						Lower	Upper
Resilience	0.002	0.962	−0.310	152	0.757	−0.210	0.153
Go-ahead	0.001	0.969	−0.382	152	0.703	−1.357	0.917
Foul play	2.65	0.105	4.20	152	<0.01	0.716	1.985
Assertiveness	0.079	0.779	0.406	152	0.689	−1.066	1.542

Knowing that Foul play factor is specific to martial arts athletes who have suffered at least one moderate injury, and in order to verity our fourth hypothesis, a binomial logistic regression was performed to examine to what extent the mentioned psychological variable predicts athletes’ injury severity. Table 5 contains the main data of the logistic regression analysis.

**Table 5:** Binomial logistic regressions analysis—Foul (violent) play as a predictor.

	Foul (violent) play
Omnibus test—Model	<0.01
Hosmer and Lemeshow test (*p*-value)	0.995
Nagelkerke *R*^2^	0.142
Overall percentage (Predicted—Percentage correct)	63.6
Wald test	13.428
B	0.359
SE	0.094
Odds ratio values	1.432
Confidence interval for Exp(B)	1.191–1.722

The binomial logistic regression analysis highlights that the model is significant: Omnibus test—Model, *p* < 0.01, chi-square value—*χ*^2^(1) = 17.219. Considering the Hosmer and Lemeshow goodness of fit test, the *p*-value is 0.995 (chi-square = 0.394), meaning that the model is not a poor fit. In the case of martial artists, the results for Foul (violent) play can predict injury severity during competitions. The model correctly classified 63.6% of cases (overall percentage). The effect size index (Nagelkerke *R*^2^ = 0.142) shows a moderate relation between Foul play and athletes’ injury severity. As an example, according to PRE_1 column (automatically generated in SPSS when running the logistic regression), an athlete having 12 points for Foul play has 85.7% probability of suffering a moderate and/or a severe injury during competitions, a martial arts athlete obtaining 6 points has a 42.8% probability, while with 5 points this probability decreases at 37%.

It was underlined that a low level of Foul (violent) play is linked with a decreased likelihood of moderate and/or severe injuries in martial artists.

## 4 Discussion

In the current research, martial artists registered a moderate level of resilience, a low level of Foul play, a slightly below average level for Go-ahead factor, and an average level of Assertiveness (at group level). In addition, specialized literature mentioned that martial arts athletes’ resilience (karate practitioners) was at mid-level (Kuçuk Kiliç, 2020) (BRS was used). Considering assertiveness, it was found that male athletes in individual sports (including taekwondo, judo, and wrestling) registered high values; however, only 32% of athletes, in the entire sample, were martial artists (Yetis, 2016).

In addition, the present study found that martial arts practitioners from SC and MMA, respectively, are significantly more resilient and score significantly higher on Foul (violent) play compared with athletes from GC. In other words, athletes in SC and MMA recover more easily after a failure or a difficult situation in life/sports career, while being more willing to win at any cost (even in an unethical way) compared with martial artists in GC. In this context, we find interesting results of Pedersen (2007) when the perceived likelihood to produce a career-ending injury was investigated; boxing (a SC) was more rated than wrestling (a GC). We point out that in the case of SC and MMA athletes, the score for the Foul play dimension is at a low level (at group level). In contrast, for the group of athletes from GC, the score is at a low to very low level.

In the next phase, the existing associations between injury severity, resilience, and athletes’ aggression level were investigated. A significant link between injury severity and assertiveness (in striking combat sports), respectively, between injury severity and resilience (in grappling combat sports) was observed. A higher assertiveness score is associated with a higher injury severity in SC, and a higher resilience value (athletes recover more quickly after a difficulty) is associated with a higher injury severity in GC. Assertiveness is particularly useful in interpersonal relationships, with coaches, sports psychologists, physical trainers, peers, or referees, for the quality of relationships with interdisciplinary team members (and not only), but athletes also express firmly, honestly what they feel or think, without hurting others. In competition, however, in the SC, we observe that a higher level of assertiveness predisposes athletes to injuries. In sports, “harm may be a consequence of assertive behaviors, but it is not the intent” (Pedersen, 2007). Regarding assertion, there is “(1) no intent to harm, (2) use of legitimate force, (3) no anger-unusual effort and energy expenditure” (Cox, 1990). Looking in context, we recommend that in striking combat sports, during competition/fighting, athletes should manifest a higher level of anger which appears to facilitate athletes’ performance (Terry and Slade, 1995; Wargo et al., 2007) and focus less on assertive behavior.

Resilience is essential in achieving sports performance, sports field being a stress-generating environment (Mellalieu et al., 2009), athletes having to face mental pressure specific to competitions, but also the “opponent inside” (e.g., fear of injury, of failure, different types of anxiety) (Predoiu et al., 2016). Resilience helps athletes thrive when dealing with adversities (Gonzalez-Mendez et al., 2023) and plays an important role in the injury rehabilitation process (Codonhato et al., 2018), being characterized by optimism, self-esteem, self-efficacy, and sense of control (Bonnano, 2004; Windle, 2011). It was found that resilience predicts higher participation (Wardlaw et al., 2018), persons with high resiliency better tolerating risky situations (McCleskey and Gruda, 2021). At a first view, the fact that a higher resilience score (in fact, a moderate/ slightly above average score, according to the norms) which is linked with a higher injury severity in grappling combat sports (judo and BJJ) is surprising, especially in the context of research by Klotz et al. (2017) who found that athletes without a concussion were characterized (among others) by resilience (however, footballers were investigated). We can discuss, at least in GC, about a level of resilience that can reduce the risk of injury, this level (in the present research) being slightly below average. As we specified earlier in the study, resilience is operating at multiple levels, being characterized (among others) by qualities such as optimism and self-esteem (and a higher self-esteem, together with a higher level of optimism, experienced too early in competition, can reduce athletes’ fighting capacity, increasing the risk of injury). In addition, considering the positive significant relationship between resilience and engagement in combat sports athletes (wrestlers were studied, a GC) (Pedro, 2016), and that engagement is interconnected with motivation, we can better explain our findings—the positive link between GC athletes’ resilience level and injury severity. It is known that motivation supposes direction, energy (effort, enthusiasm, and intensity), and duration toward actions, while “engagement is the visual manifest” of these components (Skinner and Pitzer, 2012). One can only think to the Yerkes–Dodson law which asserts that, in a complex task (and sport is characterized by complex tasks), a higher (or lower) level of arousal impairs performance (Chaby et al., 2015) (however, sports performance is idiosyncratic, each athlete having his/her own facilitative level of arousal and stress). Future research should shed more light on the level of resilience that relates to less severe injuries in a given sport and, at the same time, facilitates sports performance.

The results highlight also positive correlations between Foul (violent) play and Go-ahead, in the case of each sport category (SC, GC, and MMA) and between resilience and Go-ahead scores (in GC and MMA groups). As the athletes scores are higher on Go-ahead factor, this is linked with a higher resilience and, in the same time, with a higher probability of violent behaviors in competition (athlete may break the rules and, therefore, could lose points or could even be disqualified). Thus, athletes who are more resilient tend to attack, to go forward no matter what (specific aspects of the Go-ahead factor), and- also, these athletes who have set their sights on not giving up regardless of the obstacles encountered are more prone to unethical actions in sport (it should be noted that the athletes, in the case of Go-ahead factor, obtained, generally, slightly above average, moderate, or slightly below average scores). These results can also be explained through the Yerkes–Dodson law, a higher level of arousal (in this case) affecting martial arts athletes’ behavior (athlete’s actions could be outside the boundaries of the game).

On the other hand, data analysis revealed that martial arts athletes who have suffered from mild, moderate, and/or severe injuries (in the entire sample) registered higher scores for Foul (violent) play as compared with martial arts athletes who have suffered only mild/minor injuries. In the first case, the values are average/slightly below average or low, while in the case of athletes with only mild injuries, the results for Foul play are, mostly, low (according to the norms, see Makarowski et al., 2021). These data may be surprising. One might expect, at first glance, that after a moderate or even severe injury, the athlete would avoid breaking the rules of the game in order to win. However, the classical frustration–aggression hypothesis (see, for example, Berkowitz, 1989) could explain these findings, an aggressive behavior being the result of frustration. In the case of martial artists who suffered moderate and/or severe injuries, it may be the accumulated frustration due to lack of preparation and lack of participation in competitions during recovery periods (sometimes for several months), a period of declining income and perhaps even confidence in one’s own ability to perform. We also bring up the fact that athletes with a history of concussion were more physically aggressive (Gallant et al., 2018) and more impulsive (Goswami et al., 2016).

Not least, the present research investigated to what extent, Foul (violent) play predict injury severity in martial artists (this factor of aggression being specific to athletes who suffered from moderate and/or severe injuries in the 4 year period examined). In 2017, researchers underline the need for future studies (in sports and work) to measure adversity (for example, by magnitude), enhancing the possibility to make causal inferences with respect to specific process phenomena (Bryan et al., 2019). What we found is that a low level of Foul play is associated with a decreased likelihood of moderate and/or severe injuries in martial arts athletes (the link between variables was moderate). As the athletes’ Foul play score decreases (for example, from 12 to 5 points), the probability of suffering a serious injury decreases (according to the example provided, from 85.7 to 37%). Sometimes, athletes are instructed to be more aggressive during the matches/fights, even to be unscrupulous to win, which may increase the risk of injury (Makarowski et al., 2021). Athletes, coaches, sports psychologists, and parents should pay attention to the win-at-all-costs mentality. Therefore, aggression management strategies are necessary for injury prevention and martial artists’ personal development. Psychology-based counseling programs can be used such as positive self-talk, relaxation techniques, self-monitoring of emotional reactions (Gould et al., 2002; Perna et al., 2003), and breathing and meditation, especially for martial artists (Hernandez and Anderson, 2015).

Sport psychology consultants can work with injured athletes to transform injury into an opportunity for positive change, “rather than focusing on returning to preinjury level of functioning” (Wadey et al., 2019). Longitudinal studies are proposed to identify the changes that occur following a traumatic event (Gonzalez-Mendez et al., 2023). In addition, longitudinal research studies are needed to scale athletes’ variability of resilience and assertiveness over time, and these psychological variables are linked with athletes’ injury severity, more exactly: a slightly below average level of resilience (in GC—judo and BJJ) and a lower level of assertiveness (in SC—boxing, kick-boxing, taekwondo, and karate) were associated with less serious injuries during competitions. A multidisciplinary and personalized perspective to better understand athletes’ resilience and how they bounce back following adversity is proposed using apps, sensors, and algorithms “to detect warning signals in the psychological and physiological data” (Den Hartigh et al., 2022).

### 4.1 Limitations and future directions

Even if the results of the present research addressed gaps in the literature (considering the link between resilience, assertiveness, foul play, and martial artists’ injury severity, according to the sports discipline practiced), the study has some limitations.

The current research was carried out only in Romania, being unclear whether the findings would be replicated in other contexts. Considering the research design (*ex post facto* design/a retrospective study) and taking into account Pocecco’s observations (Pocecco et al., 2013) on the frequency of reported sports injuries in different context, one can argue the need for: (1) prospective studies (longitudinal investigations of athletes’ level of resilience and aggression to emphasize how these psychological variables influence athletes’ injury severity) and (2) retrospective studies (RS) based on institutional documentation (not RS utilizing surveys, the declarative data being subjected to more subjectivity and error—see Predoiu et al., 2022b for limits of explicit measures). However, self-report measures were used in many studies related to sports injury (e.g., Williams et al., 2020; Roșu, 2022), while Rădoi et al. (2019) highlighted that questionnaires “are critical tools for identifying patients with persistent post-concussion symptoms and their follow-up.”

Additionally, other factors of aggression such as anger, physical and verbal aggression, and hostility (Buss and Perry, 1992) should be examined in relation to martial arts athletes’ injury severity, as well as variables such as recovery, sleep quality, nutrition, and martial artists’ exercise capacity. Not least, implicit aggression, investigated through indirect measures (see Predoiu et al., 2022a for an Aggression IAT used in sports), can also be assessed in relation to athletes’ injury severity and maybe in the context of an even number of fights (for every martial artist).

## 5 Conclusion

The current study underlined that a low level in the case of Foul play factor is associated with a decreased likelihood of moderate and/or severe injuries in martial artists (in the entire sample). Therefore, a reduced frequency of violent game strategies to harm and block the opponent in an unethical manner (this type of aggression supposes unfair actions/play) helps to prevent more serious injuries in athletes. Additionally, it was found that a low or a slightly below average level of assertiveness is linked with a decreased probability of severe injuries in athletes from striking combat sports (karate, kick-boxing, boxing, and taekwondo), while in the case of grappling combat sports (judo and BJJ), a slightly below average level of resilience correlates with a decreased likelihood of more serious sports injuries. Thus, resilience and assertiveness represent psychological variables which play an important role in injury prevention, with respect to specific sports disciplines. It is recommended that specialists should work with athletes on aggression management, modeling factors such as foul (violent) play and assertiveness being a priority to enhance participation and for martial artists’ injury prevention.

## Data availability statement

The raw data supporting the conclusions of this article will be made available by the authors, without undue reservation.

## Ethics statement

The studies involving humans were approved by the research was conducted in accordance with the recommendations of Helsinki Declaration and approved by the Ethics Committee—National University of Physical Education and Sports, Bucharest, Romania (ID: 748/SG). The studies were conducted in accordance with the local legislation and institutional requirements. The participants provided their written informed consent to participate in this study.

## Author contributions

IP: Conceptualization, Formal analysis, Investigation, Methodology, Writing – original draft. RG: Resources, Supervision, Validation, Writing – review & editing. MB: Formal analysis, Investigation, Methodology, Resources, Writing – original draft. LV: Investigation, Methodology, Validation, Visualization, Writing – original draft. RM: Methodology, Resources, Visualization, Writing – review & editing. AB: Conceptualization, Resources, Writing – review & editing. SN: Conceptualization, Supervision, Validation, Visualization, Writing – original draft.
